# Bechgaard Salt‐Like Polymers and Their Applications in Organic Electronics

**DOI:** 10.1002/marc.202500948

**Published:** 2026-02-04

**Authors:** Bartlomiej Kolodziejczyk

**Affiliations:** ^1^ College of Engineering, Computing and Cybernetics Australian National University Acton ACT 2601 Australia

**Keywords:** bechgaard salts, conducting polymers, micro‐structures, micro‐wires, organic electronics, polythiophene, vapor phase polymerization

## Abstract

Over the last two decades, there has been substantial development in the manufacturing of nano‐ and micro‐ tubes and wires as building blocks for electronic devices, such as field‐effect transistors, to complement the conventional semiconductor transistors in electronic circuits. Previous attempts to improve device performance through the development of new materials have focused mostly on carbon or metal oxide‐based semiconducting materials. Here, we report a flexible conducting polymer micro‐wire grown from the vapor phase via an oxidative polymerization route. Electrical measurements show that the single micro‐wires have relatively high conductivity and non‐linear electrical characteristics. In addition, we demonstrate the fabrication of a flexible polythiophene micro‐wire organic electrochemical transistor, in which the channel and gate are made of single micro‐wires. These devices are fully compatible with conventional fabrication processes and operate in the sub‐volt regime, and have the potential to be scaled to larger multi‐micro‐wire architectures and circuits. This study demonstrates the concept of self‐assembly of organic molecules and simultaneous polymerization to generate complex, ordered, and functional structures resembling polymerized Bechgaard salts.

## Graphical abstract

The report presents a flexible conducting polymer micro‐wire grown from the vapor phase via an oxidative polymerization route. Electrical measurements show that the produced micro‐wires have relatively high conductivity and non‐linear electrical characteristics. The report shows the ability to use produced micro‐wires in organic electrochemical transistor applications where both channel and gate are made of single micro‐wires.

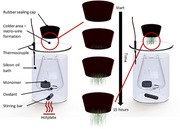

## 1 Introduction

The discovery of conducting polymers (CPs) in the 1970's opened up a new field in modern electronics, employing conducting polymers in organic electronics [1] and optical devices [2]. The main advantages of using CPs for these applications include the low manufacturing costs [3], unique electronic properties, their soft structure, flexibility, tunable functionality, and optical transparency [4, 5, 6]. Superconductivity in metals and metal‐oxides is well‐known [7] and has received much attention within the scientific community. Due to the much lower electrical conductivity of conducting polymers when compared to their metal counterparts, organic materials were not expected to exhibit superconductivity until Bechgaard observed the phenomenon in di‐(tetramethyltetraselenafulvalene)‐hexafluorophosphate ((TMTSF)_2_PF_6_) in 1979 [8]. Later, Bechgaard formed a number of different crystals using small, conjugated, and planar organic molecules exhibiting superconductivity at low temperatures [9], later known as Bechgaard salts. The macroscopic crystals of these salts were made using evaporative methods, where “monomers” stack on top of each other from the vapor phase, forming large superconducting crystals. It appears that the standard theory of superconductivity developed by Bardeen, Cooper, and Schrieffer [10] does not apply to superconducting organics due to the completely different atomic structures, and is a topic of ongoing research [11, 12]. One other interesting aspect of Bechgaard salts is that they exhibit strongly anisotropic conductivity, which can differ by multiple orders of magnitude between three crystal axes [13, 14]. Similar vapor methods are widely used to synthesize highly conductive and ordered polymers [15, 16], Vapor phase polymerization (VPP) and chemical vapor deposition (CVD) are two techniques where polymers are grown from a gaseous state. In the former, vaporized monomer condenses on the substrate that has been previously coated with oxidant so that monomers are polymerized oxidatively to form polymer chains. The latter technique differs from the first only by the fact that both the monomer and oxidant are in the vapor phase. Much of the original work on chemical vapor deposition has been done by Karen Gleason and coworkers [17, 18, 19, 20]. A recent report by Gleason, et al. has shown the ability to produce hybrid organic and inorganic nanostructures based on gallium nitrate and poly(3,4‐ethylenedioxythiophene) using oxidative chemical vapor deposition [21]. One of the latest reports by Gleason and colleagues also showed the ability to adapt chemical vapor deposition to achieve advanced morphological tunability of polymer thin films [22]. Ramli, et al. have recently reported vapor deposition as a viable pathway for the production of three‐dimensional monoliths based on various functional polymers [23]. Vapor phase techniques have several advantages over electrochemical methods in that they do not require an electrically conductive substrate, nor do they require expensive electrochemical devices to perform the synthesis, and they can deposit on virtually any surface, regardless of the shape. Attempts to employ conducting polymers and Bechgaard salts for organic electronics have attracted a large amount of attention in the scientific community due to the previously mentioned advantageous material properties. Silicon‐based devices have reached their performance limits, while limits of performance in organic materials are constantly challenged, resulting in materials of superior functionalities [24]. The emergence of the organic electrochemical transistors (OECTs) is an example of recent success that has found applications in the fields of sensors [25, 26], biology and bioelectronics [27, 28], owing largely to the novel architecture and the organic materials used to manufacture them. Here, we present a CVD method for producing Bechgaard salt‐like conducting polymers and their application in organic electronic devices, namely organic electrochemical transistors.

## 2 Discussion and Results

The polythiophene micro‐wires presented here were grown in a process similar to CVD, where both oxidizing agent and polymer precursor are evaporated [16]. The CVD processes in vacuum were also used by Bechgaard to produce his salts [9, 29]. Evaporation‐induced self‐assembly and simultaneous oxidative polymerization lead to the formation of highly ordered polythiophene materials. Terthiophene (TTh) (or bithiophene (BTh)) and toluene‐4‐sulfonic acid (PTSa) were placed in the same 500 mL flask, which was placed in an oil bath and heated to 110°C—a temperature that is above the melting point of the compounds. The vapors then travelled to the colder top of the flask, where they condensed in the form of small needle‐like crystals; this process is similar to the process used by Bechgaard. The mixture of PTSa with BTh or TTh within the crystal structure results in BTh and TTh being oxidatively polymerized. PTSa has previously been reported as an oxidant used in polythiophene synthesis [30, 31, 32]. The melting point of PTSa is 38°C and ∼103°C for PTSa•H_2_O. PTSa can decompose at elevated temperatures (> 100°C) to produce volatile toluene and sulfur trioxide [31, 32];

(1) $$\mathrm{PTSa}\cdot H_{2}O\;↔\;C_{6}H_{5}{\mathrm{CH}}_{3}+{\mathrm{SO}}_{3}+H_{2}O$$

The equilibrium of this reaction strongly favours the production of PTSa (the reaction of SO_3_ with toluene is the commercial route to produce PTSa). However, SO_3_ is known to be an oxidant toward thiophenes [31, 32, 33], itself being reduced to SO_2_;

(2) $$\mathrm{nTTh}+{\mathrm{nSO}}_{3}\;\to \;\left(\mathrm{PTTh}\right)n+{\mathrm{nSO}}_{2}+{\mathrm{nH}}_{2}O$$

While thermally initiated polymerization of thiophenes has been reported previously [34, 35], it is important to note that polymerization has not been possible without the addition of toluene‐4‐sulfonic acid (PTSa). While both BTh and TTh formed highly ordered crystals, these crystals could be easily dissolved in a range of solvents, leading to the conclusion that thermally initiated polymerization did not take place and that stacked crystals are simply made of BTh and/or TTh. Crystallinity and order interlinked with it have been considered as a pathway to enable high electrical conductivity. Recent work by Xie, et al. has shown that exceptionally high conductivity (1,200 S cm^−1^) can also be achieved in amorphous coordination polymers such as nickel tetrathiafulvalene‐tetrathiolate [36]. The same polymer material (nickel tetrathiafulvalene‐tetrathiolate) has later been shown to be an exciton condensate (a Bose‐Einstein condensate comprising pairs of particle‐hole). This report also puts a new light on the findings related to Bechgaard salts, which require high pressures and compressed geometries to achieve exceptionally high conductivity [37].

**FIGURE 1:**
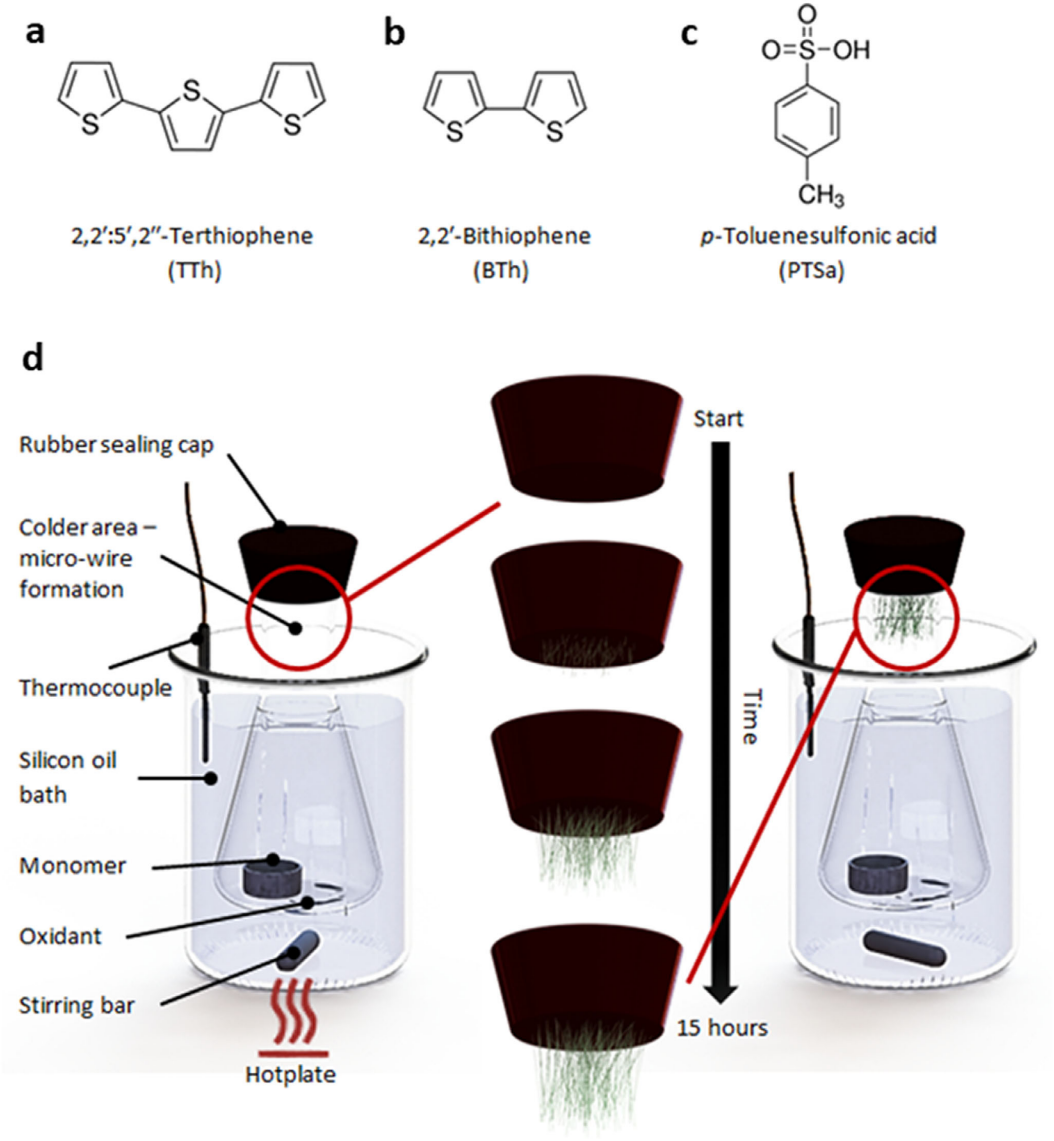
Chemical structure of used compounds, including (a) terthiophene (TTh), (b) bithiophene (BTh), and (c) toluene‐4‐sulfonic acid (PTSa). (d) Computer‐generated presentation of a simple reactor setup with steps involved in the growth of micro‐wires. PTSa and BTh (TTh) are heated up and evaporate simultaneously. Both molecules travel to the colder section of the flask (neck), condensate and form crystal‐like structures similar to Bechgaard salts. Subsequent oxidative polymerization produces highly ordered conductive polythiophene micro‐wires. The whole process takes around 15 h. Heat gradient along the polymerization chamber is shown in Figure S2.

Figure 1 shows the steps involved in the synthesis of the polythiophene micro‐wires. The as‐grown micro‐wires appear as a cloud of randomly distributed needles [Fig marc70221-fig-0002] of varying length. Washing of the resultant structure in ethanol removes unused PTSa and any unpolymerized thiophene monomers. It is commonly observed that not all PTSa is consumed during the polymerization step, as traces of it can be detected during the washing process. Similarly, pure BTh and TTh are relatively soluble in ethanol and dissolve readily along with PTSa. A control experiment was performed whereby PTSa was heated on its own to give the same needle‐like crystals. However, those crystals were found to be unstable in ambient air, and after some time shrink and dissolve due to exposure to water from the humid air. These crystals also differed in colour; PTSa crystals are white, PTSa‐polythiophene crystals are white with a hint of green, while washed polythiophene is green. It was observed that, when terthiophene or bithiophene monomers were added to the polymerization chamber at a later stage when *p*‐Toluenesulfonic acid crystals were already formed, the polymerization occurred only around the existing *p*‐Toluenesulfonic acid crystal. Upon washing, the *p*‐Toluenesulfonic acid crystals can be removed, leaving hollow polythiophene tubes as the final product (Figure S1).

**FIGURE 2 marc70221-fig-0002:**
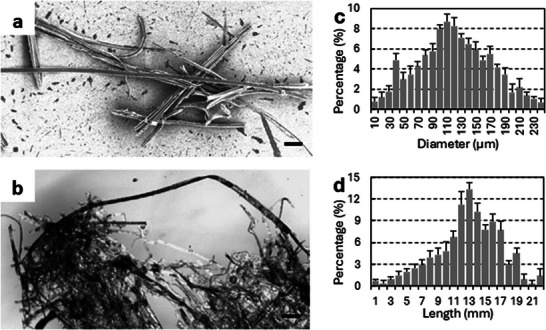
(a) SEM image of single polyterthiophene micro‐wires. The scale bar represents 50 µm. (b) Microscopic image of a bunch of polyterthiophene micro‐wires entangled together. The scale bar represents 500 µm. (c) Diameter distribution of the micro‐wires. (b) Length distribution of the micro‐wires. Both diameter and length distributions were measured from SEM images. Measurements have been repeated three times (*N = 3*) and the standard deviation calculated. Plots c and d show the mean value and positive standard deviation.

Freshly prepared, unwashed micro‐wires are rigid and brittle. Washing in ethanol makes them flexible and easy to bend and twist due to the removal of unused PTSa. Previous reports also showed that both methanol and ethanol wash led to de‐doping of polythiophene thin films [38, 39], where methanol and/or ethanol remove counter ions, resulting in polythiophene films in a semiconducting state [38]. The micro‐wires vary in diameter—ranging from 10 to 240 µm, and length—starting from 200 µm up to 2.2 cm and more, allowing synthesis of micro‐wires with a broad range of properties (Figure 2). Micro‐wires prepared in the same batch are not identical, and their dimensions may be greatly different. Geometrical parameters of the wires can be easily adjusted to meet desired requirements by varying the time and temperature of the synthesis.

**FIGURE 3:**
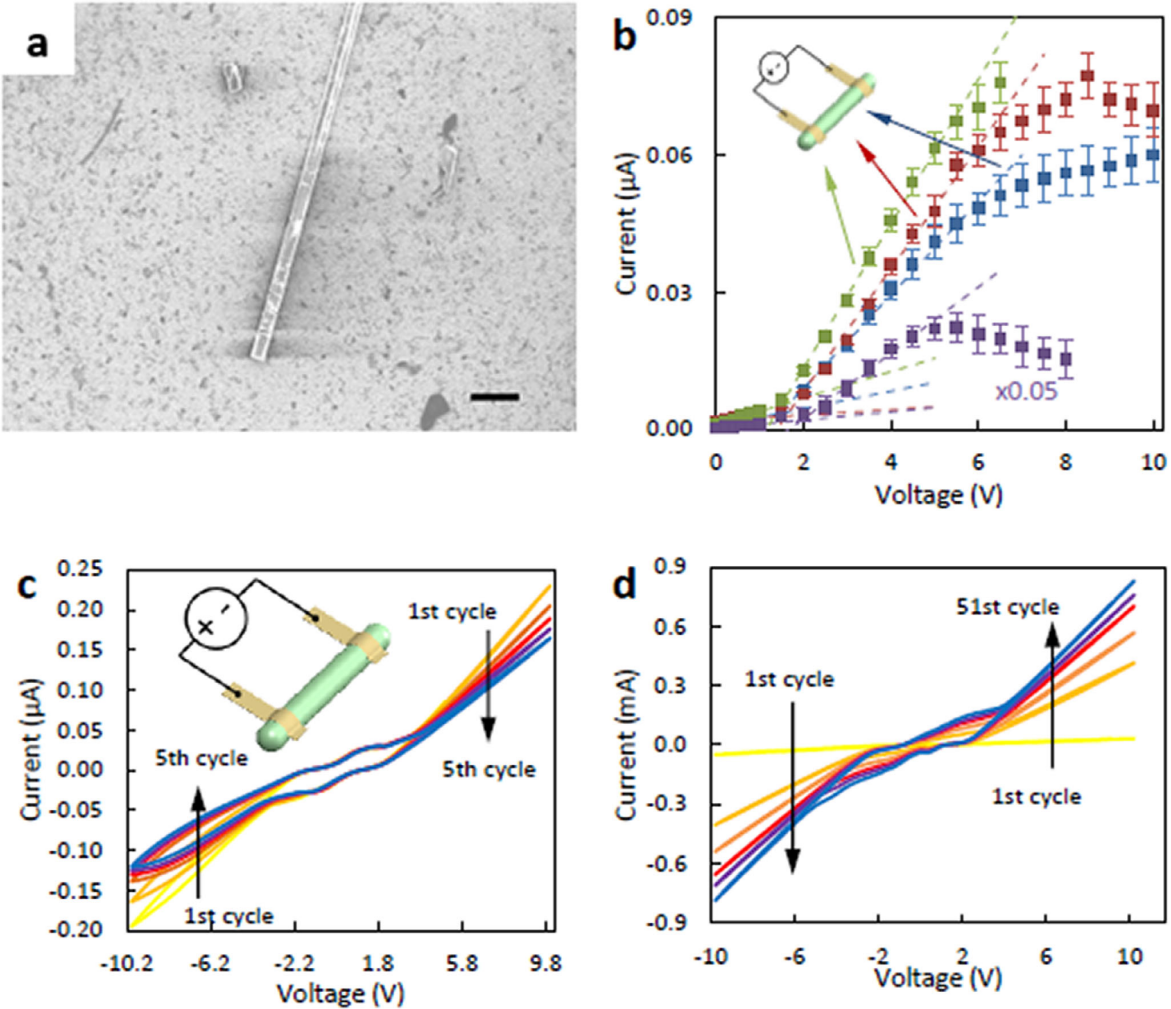
(a) Scanning electron microscope image of a single polybithiophene micro‐wire. The scale bar represents 10 µm. The visible damage on the micro‐wire due to the SEM beam, which is associated with the focusing of the beam for higher magnification images. (b) Electrical characteristics of a single polybithiophene micro‐wire in dark (blue), under illumination of 1500 W cm^−2^ (red), under illumination of 3300 W cm^−2^ (green), and a randomly ordered bunch of micro‐wires in dark (purple). The diameter of the micro‐wire was 110 µm and the distance between the contacts was 3 mm. The dimensions of the micro‐wire bunch were 2.5 mm width, 6 mm length, and 230 µm height. (c) Cyclic voltammogram performed on a single micro‐wire. The diameter of the micro‐wire was 110 µm and the distance between the contacts was 3 mm. (d) Cyclic voltammogram performed on a bunch of micro‐wires with the dimensions as mentioned above. Both cyclic voltammetry measurements were performed in two electrode setup at 50 mV s^−1^. Gold strips were used on both sides of the micro‐wire as a contact material.

**FIGURE 4:**
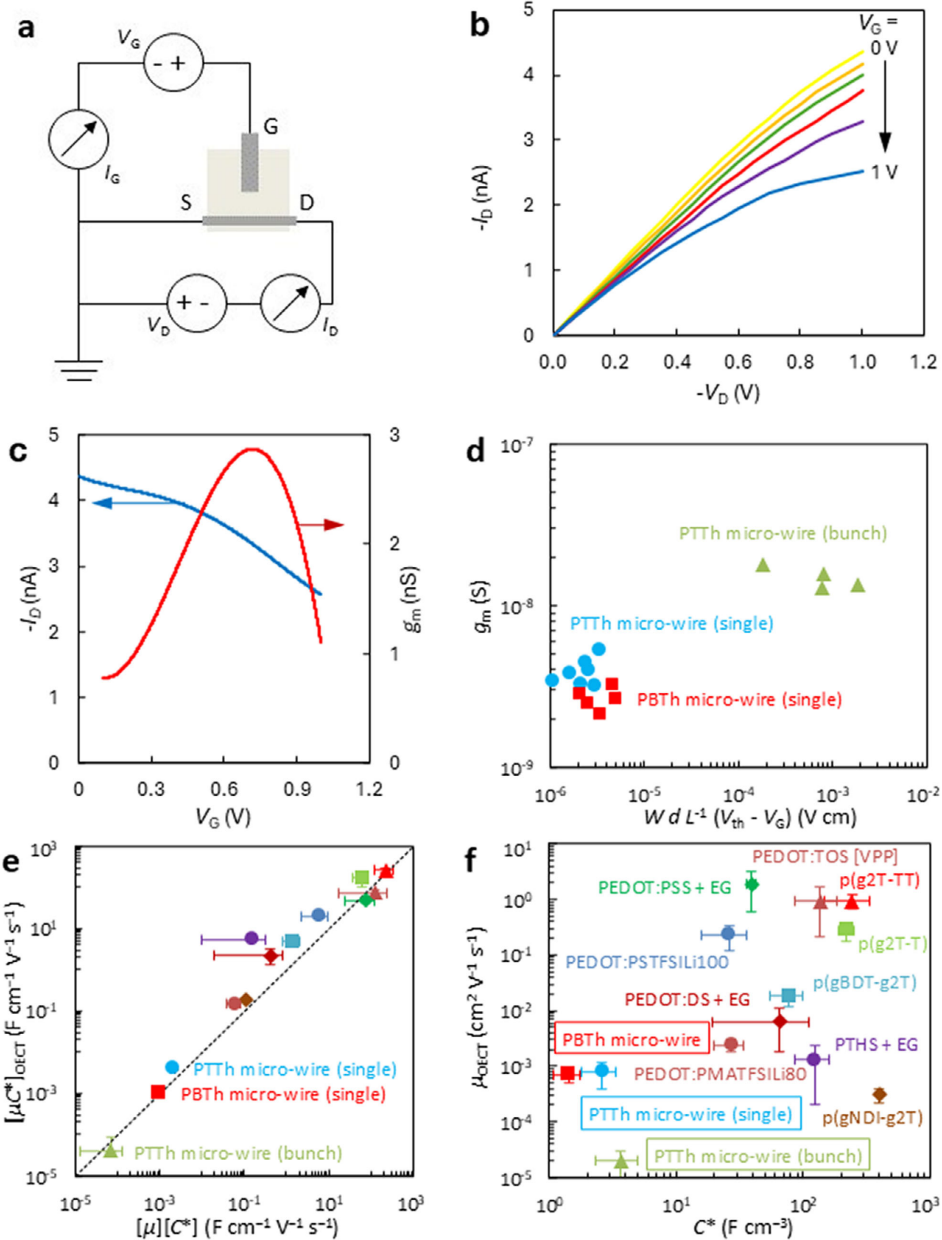
(a) Setup and circuit used to test polymer micro‐wire organic electrochemical transistor. (b) *I–V* curve for polyterthiophene polymer micro‐wire transistor. V_D_ was swept from 0 to −1 V with a step of 0.01 V for V_G_ values from 0 to 1 V with a step of 0.2 V. (c) Transfer curve and transconductance of the polyterthiophene device recorded at constant V_D_ *= *−1 V, for V_G_ ranging from 0 to 1 V. The diameter of the channel micro‐wire was 131 µm, and the distance between the contacts was 5 mm. The diameter of the gate micro‐wire was 223 µm. Transfer curve and transconductance have been smoothen using OriginPro and its Savitzky‐Golay. (d) Transconductance (*g_m_*) of micro‐wire OECTs as a function of operating conditions (in saturation regime) and channel geometry. Each point represents one OECT measurement. Color and shape represent one material, as indicated in the labelling in Figure 4f. (e) The linear slope of the data in Figure 4d, [µC^*^]*_OECT_*, as a function of the product of *µ_OECT_* and C^*^ determined independently, [*µ*][C^*^]. The dashed line represents a 1:1 agreement between the values. The color coding and shape for each material are as noted in Figure 4f. (f) *µ*_OECT_−*C* ^*^ map of Bechgaard salt‐like polymers and materials reported previously [36]. All micro‐wire experiments were performed in NaPTS solution (pH 7.1).

The new wire‐like polythiophene material was obtained for the first time, showing unique electrical and electronic properties, and therefore a range of experiments was conducted to research similarities and differences to conventional thin‐film materials. The *I–V* characteristics of single micro‐wires are presented in Figure 4b–d is indicative of semiconductor‐like behavior. This is seen as a threshold for conduction in both forward and reverse bias, due to the injection barrier or field‐dependent mobility. This behavior is typical in molecular devices. The same test was also performed while under illumination (Figure 3b), showing a decreased resistance due to the presence of light. This indicates that shining light on the material increases the conductivity of the material [40]. Similar electrical characteristics have been measured for randomly ordered micro‐wires (Figure 3b,d). Due to the random configuration of micro‐wires, the bulk structure behaves as a resistor rather than a semiconductor with a linear *I–V* relationship. The conductivity was calculated using the measured resistance values from the previous experiment, along with the dimensions of the wires measured using optical microscopy. Conductivity values of around 9·10^−4^ S cm^−1^ for a single wire were obtained, which increases to around 12·10^−4^ S cm^−1^ for micro‐wires in a randomly ordered structure. The conductivity of the wire is almost two orders of magnitude higher than the conductivity for the majority of polythiophenes synthesized chemically in aqueous media [41] However, previous report of thiophene monomer polymerization in dichloromethane with anhydrous iron chloride (FeCl_3_) solution in acetonitrile achieve electrical conductivities as high as 9 S cm^−1^ [42], while another study where polythiophene was polymerised in binary organic solvent system showed electrical conductivity reaching value of up to 20.1 S cm^−1^ [43]. Ge and colleagues have previously reported production of “metre‐long” conductive polymer wires based on poly(3,4‐ethylenedioxythiophene): poly(styrenesulfonate), which exhibit electrical conductivities as high as 1,433 S cm^−1^, and a uniform width of 50 µm [44]. However, these wires rely on different conductive polymers and have been produced in a different method, relying on the injection of a solution containing dispersed poly(3,4‐ethylenedioxythiophene): poly(styrenesulfonate) into a coagulation bath [44].

Transistor measurements using the micro‐wires taken on a dual‐channel Keithley source‐measure unit (circuit shown in Figure 4a) indicate behavior similar to OECTs described elsewhere [45, 46, 47]. Three different configurations were tested. Two configurations relied on OECTs made of two micro‐wire crystals and a larger gold strip gate electrode. The only differences between these two configurations were the materials used (PTTh or PBTh) and the geometry of the channel and gate electrodes, which is hard to control. The gate electrode was larger to provide better doping control of the channel [35]. The third configuration relied on a channel made of a bunch of randomly ordered micro‐wires which formed a larger coherent geometry, while gold was used for the gate electrode for better doping control. Attempts to produce OECT based on amorphous PTTh or PBTh thin films have failed due to poor doping/dedoping of these materials, hence the lack of comparison with amorphous materials.

The highest performing device was based on polyterthiopehene channel, providing the highest gain and largest transconductance values. The device made of a bunch of wires showed higher transconductance, but it wasn't stable over time. However, the overall performance of these structurally new materials was generally lower than the performance of PEDOT‐based OECTs (Figure 4e,f), which are considered to be model materials.

The main difference in the micro‐wire OECT performance is that the currents are much smaller compared to conventional OECTs, which can be explained by poorer conductivity. Most of the OECTs reported are based on PEDOT [20, 34, 35, 36], which is well known to have a far superior conductivity to the polythiophene micro‐wires (in the range of 5‐6 orders of magnitude). The drain current response to changes in gate voltage is shown in Figure 4b for the all‐polymer micro‐wire transistor. Doping/dedoping in this type of transistor is quite poor. The poor transconductance (Figure 4c) can be associated with the use of a larger polythiophene micro‐wire as a gate electrode rather than using a material with higher conductivity, such as gold. A gold electrode was used for a bulk micro‐wire transistor, showing much better transconductance (Figure 4d). Maximum transconductance (*g_m_*) of 3.6 ± 0.3 nS (*N = *7), 2.9 ± 0.4 nS (*N = *5), and 15.1 ± 3.4 nS (*N = *4) for respectively polyterthiophene micro‐wires, polybithiophene micro‐wires, and a bulk bunch of random polyterthiophene micro‐wires (Figure 4d) is low compared to previous OECT studies based on highly conductive PEDOT thin films [35, 37]. Volumetric capacitance (C^*^) of the materials is 2.6 ± 0.8 F cm^−3^, 1.4 ± 0.3 F cm^−3,^ and 3.6 ± 1.3 F cm^−3^ for polyterthiophene micro‐wires, polybithiophene micro‐wires, and a bulk bunch of random polyterthiophene micro‐wires, respectively (Figure 4f). The values are much lower compared to values presented by Inal et al. [36]., however these values are superior to values for amorphous polyterthiophene thin films ranging from 0.7–0.8 F cm^−3^.

Figure 4d represents the transconductance of micro‐wire OECTs as a function of operating conditions and channel geometry. The OECT based on polybithiophene micro‐wires exhibits poorer transconductance compared to the micro‐wire polyterthiophene device of similar geometry. The highest transconductance can be seen in OECTs made of a bunch of polythiophene crystals. This high transconductance is due to much larger channel geometry and the use of gold wire as the gate electrode. Highly conductive gold provides better channel doping and dedoping. Large discrepancies in OECTs based on a bunch of micro‐wires are caused by the random order of the crystals in the channel.

Figure 4e shows measurement discrepancies between material properties measured using the OECT setup ([*µC* ^*^]_OECT_) and the product of [*µ*] and [*C* ^*^] measured independently using methods described in this manuscript. The closer the points fall to the line, the lower the measurement discrepancies. A clear, nearly 1:1 correlation can be seen between [*µC* ^*^]_OECT_ and [*µ*][*C* ^*^], however values for all three studied cases remain low compared to state‐of‐the‐art benchmark materials used for OECTs.

The *µ*_OECT_−*C* ^*^ map shown in Figure 4f compares materials developed in this study to other materials used in organic electrochemical transistors due to their excellent performance. The aim of *µ*_OECT_−*C* ^*^ map is to establish a relation between volumetric capacitance and mobility of the material. For OECT applications, materials with high volumetric capacitance and high mobility are preferred [36]. The polythiophene Bechgaard salt‐like polymers show poor performance compared to the state‐of‐the‐art benchmarks, however based on the values for amorphous polythiophene thin films, these microwires show superior performance. It has to be noted that attempts to produce a functioning organic electrochemical transistor based on amorphous polythiophene thin films have thus far failed. The device channel was conductive when using polythiophene thin films, however there was no visible drain current change upon application of different gate voltages. As such, micro‐wires are more suitable candidates for organic electronic applications. Values presented in Figure 4e,f can be found in table S1 to this manuscript.

The significant difference in OECT performance of the micro‐wires compared to conventional polythiophene thin films is the much higher transconductance and current gain between on and off states. This is mainly due to the different, more crystalline structure of the material and the geometry of the device. Microwires provide a higher active surface compared to traditional flat device architecture.

**FIGURE 5:**
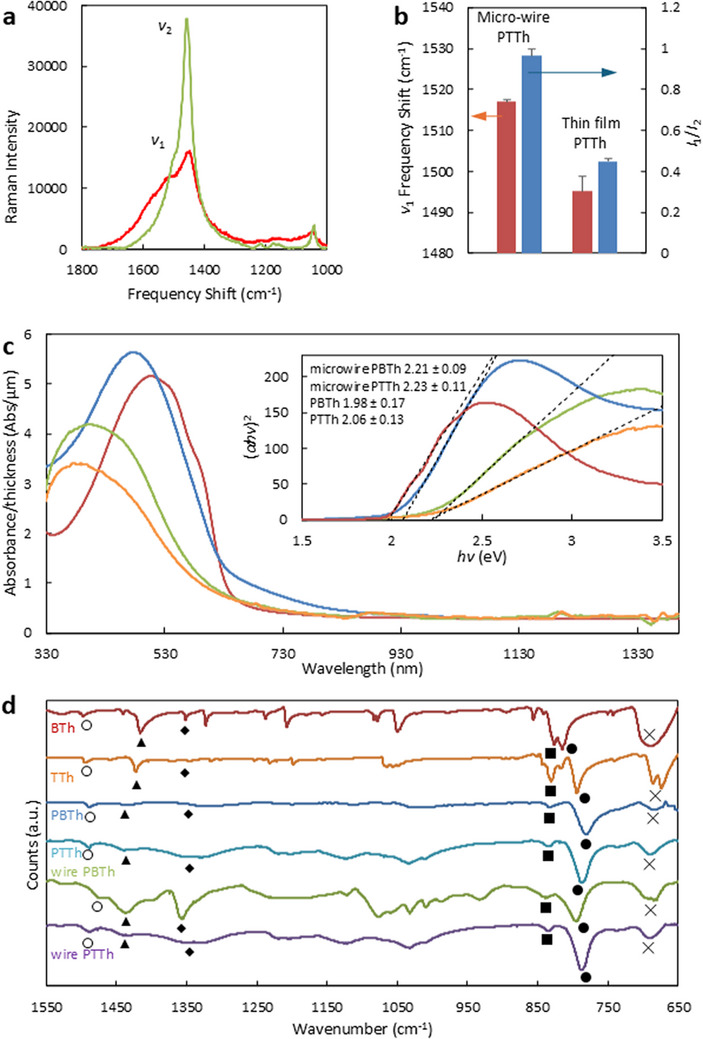
Raman spectroscopy measurements. (a) Thin film polyterthiophene sample (green) and polytherthiophene micro‐wire spectra (red) as gathered. (b) Comparison of conjugation (red) and chain lengths (blue) deconvoluted from Raman spectra. Measurements have been repeated three times (*N* = 3) and the standard deviation calculated. The plot shows the mean value and positive standard deviation. Similar comparison for PBTh can be found in Figure S3. (c) UV–vis spectra for polybithiophene (red), polyterthiophene (blue), polybiothiophene micro‐wires (orange), polyterthiophene micro‐wires (green). Estimation of band gap based on UV–vis transformation (insert), mean band gap value, and standard deviation have been calculated based on three measurements. (d) FT‐IR spectra of bithiophene and terthiophene monomers, as well as polythiophene thin films and micro‐wires based on those monomers. Symbols corresponding to specific peaks have been explained in the manuscript.

Performing Raman spectroscopy on a randomly ordered and stacked bunch of micro‐wires is a difficult task due to the irregular shape and empty spaces between wires. Raman spectra show two distinguished polythiophene peaks (Figure 5a), called *v_1_* and *v_2_*, as reported previously [48]. This is evidence of the fact that the micro‐wires are made of polythiophene. However, in samples containing micro‐wires, there is a clear frequency shift of the *v*_1_ mode from its original position. A broadening of both the *v_1_* and *v_2_* modes is also observed with an associated decrease in intensity when compared to a thin film of polyterthiophene. Peak broadening is typically a result of heat damage from laser beam irradiation, a less ordered material, or simply more variety in conjugation lengths. Uneven surfaces, like in the case of micro‐wires, can also partially diffract the laser light, thereby affecting the spectra. The *v_1_* mode is related to ring deformation of the end rings on the polymer chain, while the *v_2_* band at around 1455 cm^−1^ band is associated with the ring deformation in the central part of the polymer chain. The Raman intensity ratio between these two peaks gives an indication of the polymer chain length. Effective Conjugation Coordinate (ECC) theory also explains the frequency dispersion of the *v*_1_ mode to lower values when the polymer conjugation length is increased. Figure 5b presents a qualitative comparison of polymer chain lengths and conjugation for polyterthiophene micro‐wires and thin films through the differences in the measured *v*_1_ mode. A similar comparison has been done for a sample based on polybithiophene and can be found in the Supporting Information. The chain length of polyterthiophene present within the micro‐wires is dramatically shorter than the chain length of polyterthiophene within the film. This can be explained by the variation in the oxidizing agent. PTh chain length is very short within the micro‐wire as the *v_1_/v_2_* intensity ratio (*I*_1_/*I*_2_) is close to 1. Larger frequency values in the *v_1_* mode can be the result of either (i) shorter polymer chains, (ii) the disruption of conjugation due to rotation of thiophene rings out of co‐planarity, or (iii) mislinkages along the chain. The reasonable upshift in frequency for the micro‐wire sample is indicative of a decrease in the polymer conjugation length. Hence, the preparation method and the polymerization parameters are a powerful tool for the manipulation of the degree of conjugation and thus the electronic properties of the polymer. Raman spectra for polybithiophene thin film and polybithiophene micro‐wires can be found in the supporting information (Figure S3).

UV–vis spectra (figure 5c) of micro‐wires based on polybithiophene and polyterthiophene have similar distinct absorption peaks, similar to absorption peaks in a thin polythiophene film. However, the absorption peak is shifted to lower wavelengths as a result of lower conjugation in the polymer chains [22, 23, 24]. This is in agreement with the Raman spectroscopy (Figure 5a,b). SO_3_ used as an oxidizing agent showed previously to produce films with lower conjugation [22, 23, 24]. This is due to the much higher reduction potential of SO_3_ compared to Fe(III), resulting in a higher possibility for mislinkages in the polymer chain. Additionally, micro‐wires show a low intensity peak at around 740 nm, which is normally related to a higher oxidation level [27]. Insert to Figure 5c shows the band gap of the materials calculated from UV–vis transformation [28, 29]. The size of the band gap in polybithiophene and polyterthiophene microwires is very similar and equals 2.21 ± 0.09 and 2.23 ± 0.11 eV, respectively. The microwire band gap is slightly larger compared to the one of polybithiophene (1.98 ± 0.17 eV) or polyterthiophene (2.06 ± 0.13 eV) thin film. That would mean that microwires are less conductive since in order for an electron to jump from a valence band to a conduction band, it requires more energy for the transition.

Presented FT‐IR spectra (Figure 5d) are similar to polythiophene spectra reported elsewhere [49, 50]. The only distinguished difference between the polymeric film and micro‐wires is that the micro‐wires have a higher doping level. Doping level is related to the amplitude of the four peaks at around ∼ 1350, 1202, 1120, 1029 cm^−1^, which are contributed from C═C (♦, 1350 cm^−1^) and C‐C (1202, 1120, and 1029 cm^−1^) ring stretching vibrations.^500^ The higher doping level is most likely due to the presence of PTSa in the wires. A similar increase in doping level has also been observed in absorption spectra of the micro‐wires (Figure 5c). The vibrations at around 686 (×) and 784 cm^−1^ (●) are assigned as C_β_‐H out‐of‐plane deformations [50], 834 cm^−1^ (■) is assigned for in‐plane ring deformation [49], this is in agreement with Raman spectroscopy. Peak at 1438 cm^−1^ (▲) is contributed to C_α_ = C_β_ symmetric stretching vibration and 1489 cm^−1^ (°) is from C_α_ = C_β_ asymmetric stretching vibration [49].

Although the spectroscopic measurements of polythiophene micro‐wires above indicate lower conjugation and chain‐length than conventional thin‐films, they are not give clues to real numbers. In order to get a better understanding of the actual chain‐length, solubility experiments were undertaken. Smaller thiophene oligomers (bithiophene to the 6‐unit sexithiophene) are soluble in thiophene. However, soaking polythiophene micro‐wires in thiophene over four days did not dissolve the micro‐wires.

Usually, a low conjugation length as described above is associated with poor electrical and electronic properties. The relatively high conductivity and the ability of the polythiophene micro‐wires to perform well in OECTs, as mentioned above, suggest that the 3‐dimensional ordering/stacking is playing a crucial role in the electronic properties of the micro‐wires. It is speculated that the charge transfer between relatively short chains is promoted by the stacking in a similar way as described for the Bechgaard Salts [8, 9]. This claim is in agreement with synchrotron measurements shown in Figure S4. GIWAXS measurements have shown that polythiophene nanowalls and micro‐wires are of higher crystallinity than the regular polythiophene films. Further, the nanowalls exhibit the highest crystallinity among all three tested sample types. The polythiophene films and nanowalls are also highly aligned, while the wires have a more isotropic crystalline orientation.

## 3 Conclusions

The developed chemical vapor‐phase method combines two widely used techniques—synthesis of Bechgaard salts and oxidative chemistry—to form highly ordered conducting polymer wires with dimensions at the micro scale. We have shown how to manufacture micro‐wires of conducting polythiophene and reported on their characterization using commonly known techniques. Polythiophene micro‐wires exhibit similar conductivity when compared to similar materials, however, the conductivity does show variation when under illumination. Single micro‐wires exhibit non‐linear *I–V* characteristics, which can be considered as an advantage for use as electrical valves in organic circuits. The length and diameter of the wires can be tuned to suit application requirements by adjusting the time and temperature of the manufacturing procedure. Lastly, we have shown how to build an organic field‐effect transistor using synthesized micro‐wires, where one wire acts as a source‐drain and another as a gate electrode. Experiments performed on the transistor show previously reported behavior, while its response to changes in gate voltage is superior compared too amorphous counterpart. Polythiophene micro‐wires can be used in future organic circuits where not only one or two micro‐wires are used, but the whole architecture is based on them. Procedures presented in this study can lead to the development of similar materials based on PEDOT. The degree of crystallinity and crystal structure of these newly derived polymerized Bechgaard salts are under further investigation.

## 4 Experimental Methods

### 4.1 Synthesis

Polymer precursors 2,2′‐bithiophene (BTh) and 2,2′:5′,2′′‐terthiophene (TTh) were obtained from Sigma–Aldrich. Oxidant, toluene‐4‐sulfonic acid monohydrate, was obtained from Merck. Preparation of the thin films has been described elsewhere [48]. Synthesis has been performed in a beaker immersed in a silicon oil bath and heated up to 110°C. The temperature was controlled using a thermocouple feedback loop. PTSa and TTh (or BTh) have been added to the beaker in small plastic cups when the desired temperature has been reached. The synthesis was run for 18 h. Produced micro‐wires have been carefully transported to a Petri dish, cooled down to room temperature, separated into single wires, washed for 8 h in ethanol, and left in the solution ready for use when needed.

### 4.2 Conductivity Measurements

Conductivity measurements were performed using a simple setup with two gold connectors at the ends of the wire. Silver paint has been applied to the connectors to ensure better contact with the micro‐wire and sandwiched between two glass slides. Bio‐Logic VMP3 multichannel potentiostat has been used to apply a constant potential between two ends of the wire and measure the resulting current for a period of 40 s, which was later averaged and plotted vs. potential. Resistance has been found by using the voltage/current slope, and conductivity has been calculated knowing resistance, diameter, and the length of the wire. Diameter and the length have been estimated from microscopic images taken on a Nikon Eclipse ME600 microscope equipped with PixeLink PL‐A662 CMOS camera.

### 4.3 Transistor Measurement

Transistor measurements have been taken using a Keithley 2612A dual SourceMeter, which was used to bias the transistor and record the drain and gate currents. Recording was done using software written in LabVIEW. Two micro‐wires have been sandwiched parallel to each other as described for conductivity measurements, and an electrolyte applied to them. The electrolyte used in this experiment was 0.1 m NaPTS adjusted to pH 7.1.

### 4.4 Capacitance Measurements

Capacitance measurements were performed using a simple setup with two gold connectors at the ends of the wire. Silver paint has been applied to the connectors to ensure better contact with the micro‐wire and sandwiched between two glass slides. Cyclic voltammetry with sweep potential ranging from ‐10 to 10 V was applied using Bio‐Logic VMP3 multichannel potentiostat. The capacitance (*C*) was calculated using Equation 3, while volumetric capacitance (C^*^) was derived by dividing capacitance by the volume of the crystal.

(3) $$C=\frac{i}{s}$$

where *i* is the average current (Equation 4) and *s* is the potential sweep rate.

(4) $$i=\frac{1}{b-a}\int_{a}^{b}I\left(E\right)dE$$

where *a* and *b* are lower and upper sweep voltages, respectively.

### 4.5 Charge Mobility Calculation

The charge mobility (*µ*_OECT_), in saturation regime, was extracted from the device transfer characteristics, using Equation 5.

(5) $$\mu_{OECT}=\frac{2L}{W\cdot C}{\left(\frac{\partial \sqrt{I_{D}}}{\partial V_{G}}\right)}^{2}$$

The charge mobility was then used for material maps and benchmarking.

### 4.6 UV–vis Measurements

UV–vis–NIR spectroscopy was performed on a Jasco V‐670 spectrometer starting from 1400 nm, slowly (400 nm min^−1^) going down to 330 nm. Spectra have been normalized to the thickness of the film, measured using a Veeco Dektak 150 stylus profilometer. The corresponding material band gaps were calculated from UV–vis using the transformation described elsewhere [28, 29].

### 4.7 FT‐IR Measurements

FT‐IR spectroscopy was performed on a PerkinElmer FT‐IR Spectrum 100. Micro‐wires were used in bunches to gather the spectrum signature, while thin film samples were powdered to the purpose of this measurement.

### 4.8 Raman Spectroscopy

Raman spectra were obtained on a Jobin Yvon T64000 Raman spectrometer equipped with a blue 487.9 nm laser. Measurements were taken from 1000 to 1800 cm^−1^, where two bands described as *v_1_* and *v_2_* are positioned [48]. The gathered data have been treated using a baseline correction, and the mentioned peaks deconvoluted. In order to get micro‐wire spectra, a bunch of randomly ordered micro‐wires stacked on each other has been used.

### 4.9 Microscopic Images

Microscopic images were taken on a Nikon Eclipse ME600 microscope equipped with a PixeLink PL‐A662 CMOS camera.

### 4.10 Electron Microscopy

SEM images were obtained using a JEOL 7100F Field Emission Gun Scanning Electron Microscope at 5 kV for morphology study. EDX was performed at 15 kV for elemental analysis. Experiments were performed on gold sputter‐coated samples.

### 4.11 Material Benchmarking and Comparison

Material benchmarking was performed as described in the study by Inal et al. [36]. The material data for comparison was also reused from Inal's study.

### 4.12 GIWAXS Measurements

For GIWAXS experiments, polymer samples were coated onto a plasma‐treated silicon wafer substrate. GIWAXS experiments were performed on the SAXS/WAXS beamline at the Australian Synchrotron in Clayton, Victoria, at the critical angle of approximately 0.08° determined as the angle of maximum scattering intensity. Measurements were scaled to momentum transfer (*Q*) space using the measurement geometry defined by a sample to detector distance of 331.3 mm, a two‐dimensional Dectris Pilatus 1 m detector with a pixel size of 0.172 × 0.172 mm^2,^ and X‐rays of wavelength, *λ = *0.689 Å. Sample geometry, including the coordinates at which the direct X‐ray beam would intersect the detector, was determined by use of a Silver Behenate scattering standard. Two‐dimensional scattering patterns were corrected to momentum transfer axes in‐plane and out‐of‐the‐plane of the sample (the *Q_xy_* and *Q_z_* axes, respectively), resulting in the missing wedge. One‐dimensional scattering intensity along *Q_xy_* and *Q_x_* was calculated from sectors of the 2D scattering patterns centred along these axes using NIKA.

## Conflicts of Interest

The authors declare no conflicts of interest.

## Supporting information




**Supporting File**: marc70221‐sup‐0001‐SuppMat.docx.

## Data Availability

The data that support the findings of this study are available from the corresponding author upon reasonable request.
