# Supplementary material for: Bechgaard Salt‐Like Polymers and Their Applications in Organic Electronics
Source: Macromol Rapid Commun. 2026 Feb 4;47(8):e00948. doi: 10.1002/marc.202500948 (PMC13087838; doi:10.1002/marc.202500948)
Supplement: Supplementary file 1 — Supporting File: marc70221‐sup‐0001‐SuppMat.docx. [file MARC-47-e00948-s001.docx]

**Bechgaard Salt-like Polymers and Their Applications in Organic Electronics**

*Bartlomiej Kolodziejczyk*

College of Engineering, Computing and Cybernetics, Australian National University, 108 North Road, Acton, ACT 2601, Australia.

Corresponding author. E-mail: kolodziejczyk.bartlomiej@gmail.com

**Keywords:** Conducting Polymers, Bechgaard Salts, Polythiophene, Micro-structures, Vapor Phase Polymerization, Micro-wires, Organic Electronics

**Hollow micro-tubes**

It has been observed that if terthiophene or bithiophene monomers are added to the polymerization chamber at a later stage when *p*-Toluenesulfonic acid crystals are initially formed, the polymerization occurs only around the existing crystal without crystal penetration. Upon washing remaining *p*-Toluenesulfonic acid is removed leaving hollow formations. While it may not be clear from SEM images below, the collages ribbon like structures are different to previously presented micro-wires. The collapse ribbon-like structures are due to their hollow nature, where polythiophene was polymerised only around *p*-Toluenesulfonic acid crystals forming hollow polythiophene tubes.

| 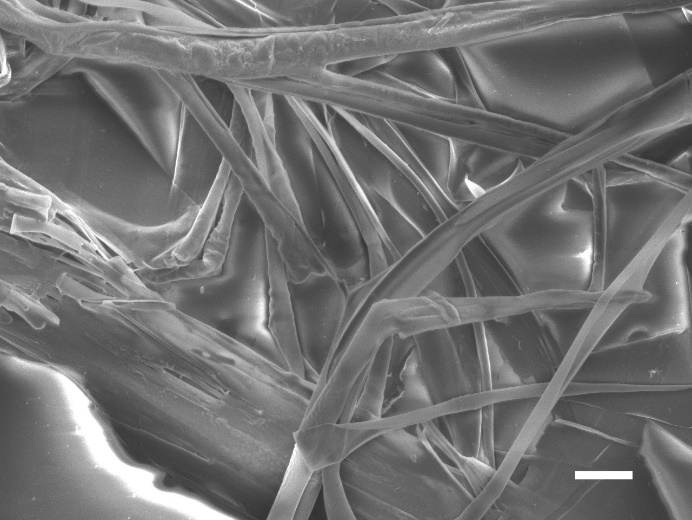 | 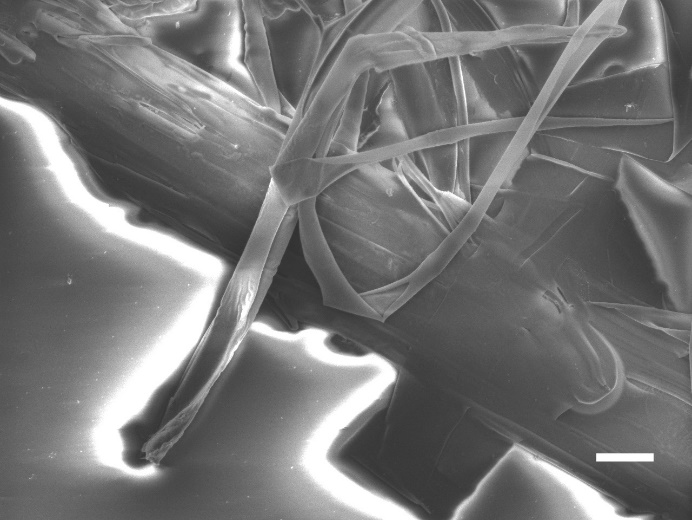 |
| --- | --- |

**Figure S1** SEM images hollow polythiophene micro-tubes. Polyterthiophene micro-tubes polymerized at 120 ºC (left) and polyterthiophene micro-tubes polymerized at 110 ºC. Scale bar in both images represents 10 µm.

**Heat distribution in polymerization chamber**

Temperature varies along the polymerization chamber. Down at the bottom of the silicon bath temperature is around 108° C. Going up; close to the oxidizing agent and monomer temperature close to 105° C, allowing evaporating PTSa and monomer. At the very end of polymerization beaker temperature ranges from 65 to 68° C, which is slightly lower than melting point of terthiophene and much lower than temperature required for decomposition of PTSa (eq 1). This low temperature allows formation of PTSa/polythiophene crystalline structures which are then polymerized (eq 2). Heat gradient in polymerization chamber has been shown on Figure S1. The temperature has been measured using thermocouple connected to measuring device.


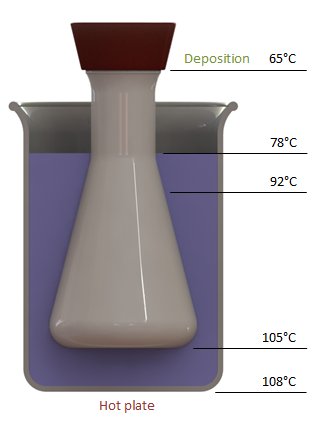


**Figure S2.** Heat gradient in polymerization chamber.

**Raman spectra for polybithiophene micro-wires**

Raman spectroscopy preformed on polybithiophene thin films and micro-wires, show similar pattern to pattern described in the main manuscript for polyterthiophene. Micro-wire *v_1_* mode is very broad and low in intensity. This can be again associated with material damage, less ordered structure or more variety. There is also significant fluorescence for micro-wire sample, which in some cases can be related to heat damage.

|  |  |
| --- | --- |

**Figure S3.** Raman spectroscopy measurements. (**a**) Thin film polybithiophene sample (green) and polybithiophene micro-wire spectra (red) as gathered. (**b**) Comparison of conjugation (yellow) and chain lengths (blue) deconvoluted from Raman spectra.

**Overheating during VPP**

It has been found that too high polymerization temperature (120 ºC and above) can result in carbonization of the micro-wire. Carbonized micro-wires are much wider in diameter than regular micro-wires. They are also very rigid and fragile. We expect that at higher temperatures different reaction occurs, possibly further PTSa decomposition, where products of this decomposition react with thiophen monomer, causing carbonization of the material. The effect can be seen on figure S3a. This gives us very narrow temperature gap for tuning the properties of the wires. We need to reach at least 103 ºC to melt and evaporate PTSa, but not more than 120 ºC to prevent carbonization. It has to be mentioned that carbonized wires can also be interesting in scientific point of view and further characterization has to be done.

**Table S1.** Materials figure of merit for various OECT materials.^1^

| Material/Formulation | *C** (F cm^−3^) | *µ* _OECT_ (cm^2^ V^−1^ s^−1^) | [*µ* _OECT_][*C**]  (F cm^−1^ V^−1^ s^−1^) | [*µC**]_OECT_ (F cm^−1^ V^−1^ s^−1^) |
| --- | --- | --- | --- | --- |
| p(g2T-TT) | 241 ± 94 | 0.94 ± 0.25 | 227 ± 107 | 261 ± 29 |
| p(g2T-T) | 220 ± 30 | 0.28 ± 0.1 | 62 ± 24 | 167 ± 65 |
| PEDOT:TOS [VPP] | 136 ± 50 | 0.93 ± 0.72 | 126 ± 108 | 72 ± 14 |
| PEDOT:PSS + EG | 39 ± 3 | 1.9 ± 1.3 | 75 ± 51 | 47 ± 6 |
| PEDOT:PSTFSILi100 | 26 ± 10 | 0.23 ± 0.11 | 6.1 ± 3.8 | 20 ± 1.6 |
| PTHS + EG | 124 ± 38 | 0.0013 ± 0.0011 | 0.16 ± 0.15 | 5.5 ± 0.1 |
| p(gBDT-g2T) | 77 ± 23 | 0.018 ± 0.006 | 1.4 ± 0.6 | 4.8 ± 0.7 |
| PEDOT:DS + EG | 65 ± 46 | 0.0064 ± 0.0046 | 0.42 ± 0.4 | 2.2 ± 0.9 |
| p(gNDI-g2T) | 397 | 0.00031 ± 0.00009 | 0.12 | 0.18 ± 0.01 |
| PEDOT:PMATFSILi80 | 27 ± 7 | 0.0024 ± 0.0006 | 0.06 ± 0.02 | 0.15 ± 0.01 |
| PTTh micro-wire (bunch) | 3.64 ± 1.3 | 0.00001 ± 0.00001 | 0.000073 ± 0.00006 | 0.00004 ± 0.00005 |
| PTTh micro-wire (single) | 2.6 ± 0.78 | 0.0008 ± 0.0004 | 0.0021 ± 0.0003 | 0.004 ± 0.0004 |
| PBTh micro-wire (single) | 1.42 ± 0.34 | 0.0007 ± 0.0002 | 0.001 ± 0.00007 | 0.001 ± 0.0002 |

Three last records (light red) correspond to materials developed in the current study. Remaining records are from another study by Inal, et al.^1^

**Synchrotron GIWAXS measurements**


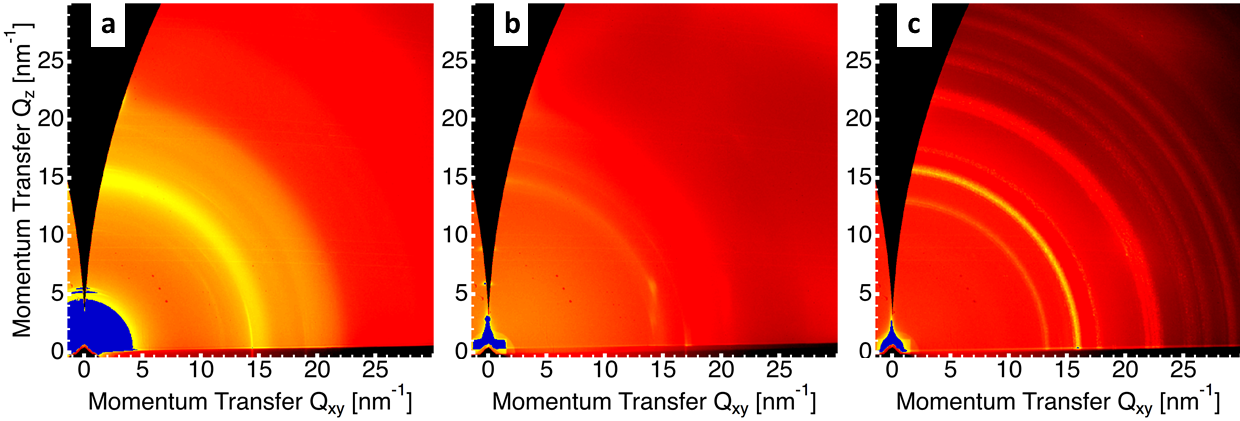


**Figure S4.** GIWAXS measurements performed at Australian Synchrotron. 2D corrected data of **(a)** polybithiophene thin film, **(b)** polyterthiophene thin film, and **(c)** bunch of polythiophene microwires.

**References**

1. Inal, S., Malliaras G. G. & Rivnay J. Benchmarking organic mixed conductors for transistors. Nature Communications 8, 1767, doi:10.1038/s41467-017-01812-w (2017).
